# Fabrication of PEDOT: PSS-PVP Nanofiber-Embedded Sb_2_Te_3_ Thermoelectric Films by Multi-Step Coating and Their Improved Thermoelectric Properties

**DOI:** 10.3390/ma13122835

**Published:** 2020-06-24

**Authors:** Sang-il Kim, Kang Yeol Lee, Jae-Hong Lim

**Affiliations:** 1 Department of Materials Science and Engineering, University of Seoul, Seoul 02504, Korea; sang1.kim@uos.ac.kr; 2 Department of Materials Science and Engineering, Gachon University, Seoungnam 13120, Korea; k9876047266@gachon.ac.kr

**Keywords:** thermoelectric, carrier filtering effect, Sb_2_Te_3_, PEDOT:PSS

## Abstract

Antimony telluride thin films display intrinsic thermoelectric properties at room temperature, although their Seebeck coefficients and electrical conductivities may be unsatisfactory. To address these issues, we designed composite films containing upper and lower Sb_2_Te_3_ layers encasing conductive poly(3,4-ethylenedioxythiophene):poly(4-styrenesulfonate) (PEDOT:PSS)- polyvinylpyrrolidone(PVP) nanowires. Thermoelectric Sb_2_Te_3_/PEDOT:PSS-PVP/Sb_2_Te_3(ED)_ (STPPST) hybrid composite films were prepared by a multi-step coating process involving sputtering, electrospinning, and electrodeposition stages. The STPPST hybrid composites were characterized by field-emission scanning electron microscopy, X-ray diffraction, ultraviolet photoelectron spectroscopy, and infrared spectroscopy. The thermoelectric performance of the prepared STPPST hybrid composites, evaluated in terms of the power factor, electrical conductivity and Seebeck coefficient, demonstrated enhanced thermoelectric efficiency over a reference Sb_2_Te_3_ film. The performance of the composite Sb_2_Te_3_/PEDOT:PSS-PVP/Sb_2_Te_3_ film was greatly enhanced, with *σ* = 365 S/cm, *S* = 124 μV/K, and a power factor 563 μW/mK.

## 1. Introduction

Because of their attractive intrinsic thermoelectric properties at near room temperature, antimony telluride (Sb_2_Te_3_) thin films have been intensively studied by several research groups [1,2,3,4]. However, the Seebeck coefficient (*S*) of Sb_2_Te_3_ at room temperature is typically low (38–83 μV/K) because of the presence of many anti-site defects resulting from the similar electronegativities of Sb and Te [5,6]. Even though an amorphous Sb_2_Te_3_ phase fabricated via an electrochemical process possessed a relatively high *S* of ~500 μV/K at room temperature, it only exhibited very low electrical conductivity (*σ*, ~10^−2^ S/cm) [5]. Therefore, to overcome this problem, scientists have modulated various factors, including chemical composition [7], crystal phase [5,8], crystallinity [9], charge-carrier concentration [10], and mobility [11,12].

The thermoelectric conversion efficiency of a material can be evaluated by a dimensionless thermoelectric figure of merit, *zT*, defined as (*S*^2^*σ*/*κ*) *T* (where *κ* is the total thermal conductivity and *T* is the absolute temperature). Therefore, increasing *S* while maintaining an optimum *σ* value, which increases the power factor (*S*^2^*σ*), is a key approach for achieving high efficiency thermoelectric materials. One strategy for accomplishing this has been the fabrication of inorganic-organic hybrid composites [13,14,15,16]. To date, researchers have investigated organic-inorganic composites such as poly(3,4-ethylenedioxythiophene):poly(4-styrenesulfonate) (PEDOT:PSS)/graphene [17], PEDOT:PSS/Te nanorods [18], PEDOT:PSS/Sb_2_Te_3_ [19], PEDOT:PSS/PbTe [20], PEDOT:PSS/Bi_2_Te_3_ [21], PEDOT:PSS/Ge [22], P3HT/Bi_2_Te_3_ [23], and polyaniline (PANI)/carbon nanotubes (CNTs) [24] to achieve high power factors. Among conductive organic polymers, PEDOT:PSS has a relatively high *σ* and environmental stability. Even though the *S* value of PEDOT:PSS is somewhat low compared with inorganic materials, it is relatively non-toxic, abundant, and exhibits a low *κ*, which would offer a higher *zT* [25,26,27]. With respect to preparing organic-inorganic composites, most methods involve simple mixing of the materials (typically solid inorganic phases and liquid organic polymers). Then, the mixture is cast onto a formal substrate prior to measuring thermoelectric properties. In these cases, the thermoelectric properties (*S* and *σ*) of the composite materials are described according to the percolation effect in the organic-inorganic system [25,28,29].

Recently, there has been significant interest in the electrospinning technique as a large-scale synthesis method for 1D organic polymer fibers with nano-sized diameters [30,31]. As electrospinning can be adapted to produce PEDOT:PSS nanofibers, herein, we suggest a new type of inorganic–organic hybrid composite material that includes 1D nanofibers of PEDOT:PSS embedded in Sb_2_Te_3_ for high power factors. To fabricate the hybrid nanofiber composite composed of Sb_2_Te_3_ and the conducting PEDOT:PSS polymers, their durability must be improved prior to use as free-standing structures over large areas. Thus, a novel method for fabricating hybrid composites is necessary to achieve good mechanical strength and thermoelectric properties. In this study, inorganic-organic-inorganic composite films were fabricated by a stepwise coating process, with sequential deposition of Sb_2_Te_3_, PEDOT:PSS, and Sb_2_Te_3_ by sputtering, electrospinning, and electrodeposition, respectively.

## 2. Experimental Section

**Figure 1:**
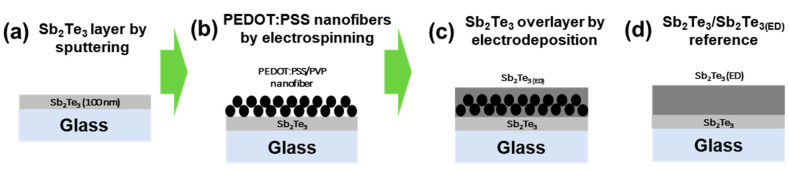
(**a**–**c**) Schematic illustration of fabrication process for PEDOT:PSS-embedded Sb_2_Te_3_ films composed of sequentially coated Sb_2_Te_3_ (sputtering), PEDOT:PSS-PVP (electrospinning), and an Sb_2_Te_3_ overlayer (electrodeposition, ED). (**d**) A reference sample without PEDOT:PSS-PVP was also fabricated.

Figure 1 schematically depicts the stepwise fabrication process for the inorganic-organic-inorganic hybrid composite films, which includes (1) deposition of a 100 nm thick Sb_2_Te_3_ thin film by sputtering, (2) coating of a layer of PEDOT:PSS-polyvinylpyrrolidone (PVP) nanofibers by electrospinning, and (3) electrodeposition of an Sb_2_Te_3_ overlayer.

### 2.1. Deposition of 100 nm Thick Sb_2_Te_3_ Thin Film by Sputtering

First, a Sb_2_Te_3_ sublayer was deposited on a glass substrate with 100 nm thickness by sputtering under vacuum conditions (~1 Pa) using the DC sputtering method with 2 inch readymade Sb_2_Te_3_ targets of 99.999% purity (Kurt J. Lesker Company, Jefferson Hills, PA, USA) at room temperature (Figure 1a). Prior to deposition, the glass substrate was polished with sandpaper (800 grit) and ultrasonically cleaned to obtain good adhesion and avoid contamination. The target was cleaned for 5 min just before deposition to remove any surface contaminants. Then, the films were annealed at both 373 and 473 K for 30 min.

### 2.2. Coating of PEDOT:PSS-PVP Nanofiber Layer by Electrospinning

Polyvinylpyrrolidone (PVP, 0.067 g) was dissolved in PEDOT:PSS solution (2.560 g) with stirring at room temperature. Then, dimethylformamide (DMF, 0.345 g) was added to the prepared solution and the mixture agitated for 24 h at room temperature to obtain a homogeneous, spinnable solution. The as-prepared precursor solution was loaded into a 5 mL plastic syringe and electrospun with an electrospinning system (ESDR200H, NanoNC Co., Seoul, Korea) from a stainless steel needle at a voltage of 10 kV and constant flow rate of 0.2 mL/min. The electrospinning process was conducted at the constant temperature and relative humidity of 25 °C and 20% ± 5%, respectively. The distance between the tip of the needle and collector was 9 cm. The electrospun PEDOT:PSS-PVP nanofibers were coated on the as-prepared Sb_2_Te_3_/glass substrate at 1000 rpm for 60 s (Figure 1b). The deposited two-layer films were stabilized at 150 °C for 30 min.

### 2.3. Electrodeposition of Sb_2_Te_3(ED)_ Overlayer to Form the Composite Film

The final layer of the Sb_2_Te_3_/PEDOT:PSS-PVP/Sb_2_Te_3(ED)_ composite film was potentiostatically electrodeposited (Figure 1c). The electrolyte was prepared by separately dissolving TeO_2_ (2.4 mM), Sb_2_O_3_ (3.6 mM), and L-tartaric acid (33 mM) in HNO_3_ solution (1 M) at 60 °C. The resulting solution was mixed and diluted to a final volume of 1 L with deionized water. The working electrode was the Sb_2_Te_3_/PEDOT:PSS-PVP film on the glass substrate. Pt and Ag/AgCl electrodes were used as the counter and reference electrodes, respectively. While maintaining the electrolyte at 25 °C and magnetically stirring at 300 rpm, a potential of −0.34 V (vs. Ag/AgCl) was applied. The deposited composite films were annealed at 373 and 473 K for 30 min.

To explore the influence of the embedded PEDOT:PSS-PVP nanofibers in the Sb_2_Te_3_ film, a reference Sb_2_Te_3_/Sb_2_Te_3(ED)_ film was fabricated for comparison (Figure 1d). The total thicknesses of the composite and reference films were regulated to be 2 μm.

### 2.4. Characterization

Scanning electron microscopy (SEM) images were obtained using a field-emission scanning electron microscope (FE-SEM, TESCAN Model MIRA 3 LM, Kohoutovice, Czech Republic). X-ray diffraction (XRD) patterns were measured with a Bruker D8 ADVANCE A25 diffractometer using Cu *K*_α_ (λ = 1.5406 Å) radiation. Fourier transform infrared (FT-IR) spectra were collected using a Thermo Fisher Scientific Nicolet iS10 spectrometer. The work function of the PEDOT:PSS wires was characterized by ultraviolet photoelectron spectroscopy (UPS, Thermo Fisher Scientific K-Alpha, Waltham, MA, USA). X-ray photoelectron spectroscopy (XPS) measurements were also carried out with the K-Alpha spectrometer, using Al *K*_α_ X-rays (1486.6 eV) as the light source. The base pressure of the chamber was ~1 × 10^−8^ Pa, and the electron takeoff angle was 90°.

### 2.5. Electrical and Thermoelectric MeasFurements

Electrical and thermoelectric properties were measured in the direction parallel to the film using a *S* measurement setup (homemade) at room temperature. This measurement system was calibrated using several materials (i.e., Al, Pt, Chromel, and Sb_2_Te_3_) and the results were compared with those obtained from commercial ZEM-3 (Advanced-riko, Yokohama, Japan). A deviation of <5% was obtained, thereby indicating that our measurement system was sufficiently accurate. The electrical resistivity of the sample was measured by a standard four-point probe method with the film size of 10 mm × 10 mm. The *S* was measured by a static dc method based on the slope of the voltage versus temperature-difference curves at room temperature as one side of the sample was heated with a heater.

## 3. Results and Discussion

**Figure 2:**
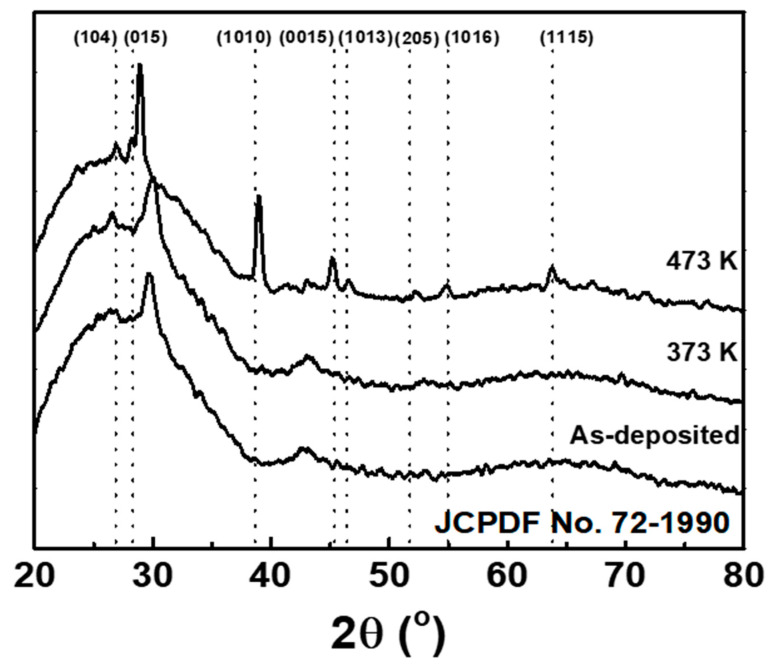
X-ray diffraction patterns of as-deposited Sb_2_Te_3_ (100 nm thickness) film and after annealing at 373 or 473 K.

The XRD patterns of the as-grown and annealed Sb_2_Te_3_ sublayer films are shown in Figure 2. Eight major diffraction peaks between 20° and 65° for the rhomb-centered rhombohedral structure of Sb_2_Te_3_ (JCPDF No. 72-1990) are indicated with dotted lines, including the (1 0 4), (0 1 5), (1 0 10), (0 0 15), (1 0 13), (2 0 5), (1 0 16), and (1 1 15) planes. The XRD patterns of all the Sb_2_Te_3_ thin films deposited on glass substrates also show broad bands (2*θ* = 20°–40°) associated with amorphous silica. The as-deposited film displays only one intrinsic diffraction peak near 30°. This XRD pattern hardly changes after annealing at 373 K. However, after annealing at 473 K for 30 min, the eight major diffraction peaks appear. This implies that the dominant metastable phase of Sb_2_Te_3_ was transformed from an amorphous structure to a crystalline state by heat treatment near 473 K for 30 min, while the nanofiber-embedded film structure was retained.

**Figure 3:**
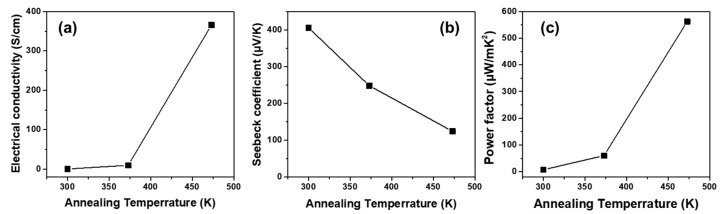
(**a**) Electrical conductivity, (*σ*), (**b**) Seebeck coefficient, (*S*), and (**c**) power factor as functions of annealing temperature (373, 473 K) for 100 nm thick Sb_2_Te_3_ films deposited on polished glass substrates at room temperature (300 K).

Figure 3 shows the *S*, *σ*, and power factor values for the as-deposited and heat-treated Sb_2_Te_3_ films as functions of the annealing temperature. The *σ* for the Sb_2_Te_3_ film is greatly enhanced from 0.45 to 365 S/cm, while the *S* decreases gradually from 406 to 124 μV/K as the annealing temperature increases to 473 K. As the crystallinity appears with the annealing process seen in XRD patterns in Figure 2, the *σ* increased. The correlation between the crystallinity of Sb_2_Te_3_ films and the *σ* was clearly shown. As a result, a power factor value of 563 μW/mK was achieved after 473 K annealing. The annealing process effectively induces crystallization of the Sb_2_Te_3_ phase, as also observed in XRD, which enhances the *σ* and power factor of the films.

**Figure 4:**
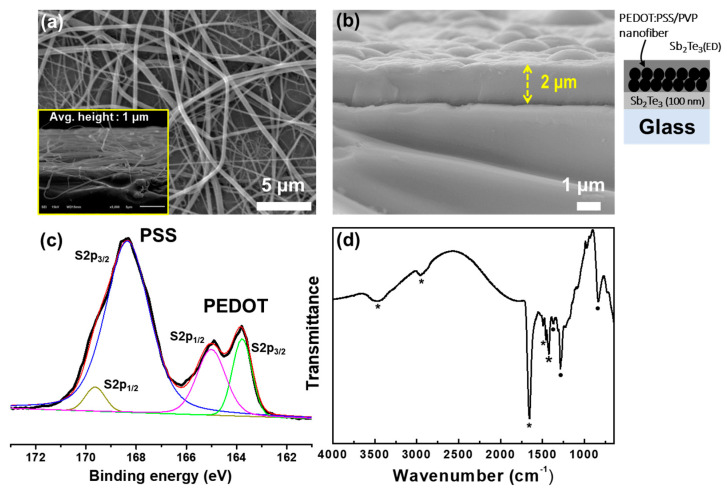
SEM of the (**a**) top view of the Sb_2_Te_3_/PEDOT:PSS-PVP composite film and (**b**) cross-sectional view after fully compositing the Sb_2_Te_3_/PEDOT:PSS-PVP/Sb_2_Te_3(ED)_ film. (**c**) XPS spectrum (black line) and deconvoluted S 2*p* peaks for the PEDOT:PSS-PVP fibers. (**d**) FT-IR spectrum of the Sb_2_Te_3/_PEDOT:PSS-PVP/Sb_2_Te_3(ED)_ composite film.

Figure 4a shows the empty spaces between the PEDOT:PSS-PVP fibers after coating by electrospinning, which would seriously deteriorate the *S* and *σ* values of the coating layer. However, the subsequent electrodeposition of the Sb_2_Te_3(ED)_ layer fills the spaces completely, as shown in Figure 4b. Thus, the inorganic-organic-inorganic composite film, Sb_2_Te_3_/PEDOT:PSS-PVP/Sb_2_Te_3(ED)_ (STPPST), with a thickness of ~2 μm was successfully fabricated by the successive multi-step coating process via sputtering, electrospinning, and electrodeposition.

XPS provides detailed information on the chemical and electronic states of a surface using only those photoelectrons that escape the material without consideration of those that undergo inelastic scattering. To determinate the XPS spectrum of the PEDOT:PSS nanofibers, the energy scale was calibrated against the C 1*s* carbon peak at 284.6 eV. The typical S 2*p* core-level spectrum of the PEDOT:PSS fibers is shown in Figure 4c. The intrinsic binding states in the S 2*p* spectrum and the corresponding peaks deconvoluted as Gaussian-Lorentzian components are shown in Figure 4c. The high-resolution XPS spectrum of S 2*p* in Figure 4c shows two groups of spin-split doublets, corresponding to S 2*p*_1/2_ and S 2*p*_3/2._ The high intensity peaks between 169.63 eV (S 2*p*_1/2_) and 168.35 (S 2*p*_3/2_) correspond to the spin-split components of the sulfur atoms in the PSS chains. Additionally, the two smaller peaks at 165.01 eV (S 2*p*_1/2_) and 163.78 (S 2*p*_3/2_) can be assigned to the sulfur atoms in the PEDOT fragments. These results are in good agreement with previous reports [32,33].

Figure 4d shows the FT-IR spectrum for the STPPST composite film (Figure 4b) in the range of 4000−650 cm⁻^1^. The observed bands can be assigned to the characteristic vibrational modes of the PVP and PEDOT:PSS components. A remarkable feature of the IR spectrum is a strong band at 1657 cm⁻^1^, assigned as the amide carbonyl stretching absorption of PVP that is typically positioned between 1695 and 1615 cm^−1^ [34]. Other bands in this spectrum appear at 1424 and 1461 cm^−1^ which result from vibrations of the tertiary nitrogen of the PVP moiety (indicated with *). The bands at 2952 and 3456 cm^−1^ are assigned to C–H and–OH symmetric stretching vibrations, respectively. Peaks at 1286 and 834 cm^−1^ are related to C–C and C–S stretching vibrations in the thiophene ring (indicated with dot symbols [35], and bands at 1371 cm^−1^ can be assigned to S = O asymmetric stretching vibrations [36]. Therefore, the FT-IR spectrum of the STPPST composite indicates that the main organic structures of PEDOT:PSS-PVP were well preserved in the STPPST composite after annealing at 473 K.

**Figure 5:**
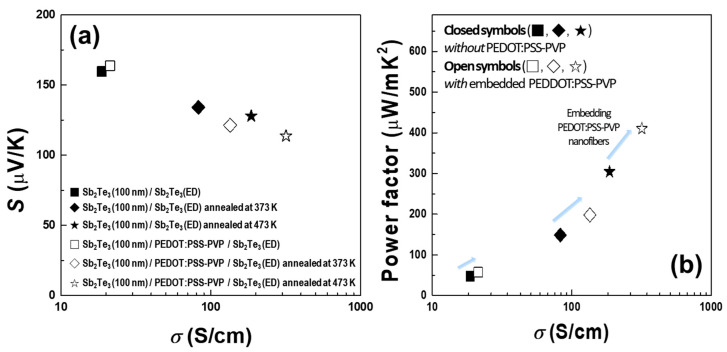
Comparison of the (**a**) Seebeck coefficients *S* and (**b**) power factors for reference Sb_2_Te_3_/Sb_2_Te_3(ED)_ and Sb_2_Te_3_/PEDOT:PSS-PVP/Sb_2_Te_3(ED)_ composite films as functions of the electrical conductivity *σ* and annealing temperature.

The *σ*, *S*, and power factor values for the reference Sb_2_Te_3_/Sb_2_Te_3(ED)_ and STPPST composite films with different annealing conditions are shown in Table 1, and the *S* and power factors as functions of *σ* are plotted in Figure 5a,b, respectively. The data clearly show enhancement of the power factor regardless of the annealing conditions by embedding the PEDOT:PSS-PVP nanofibers in Sb_2_Te_3_. In the samples without annealing, *σ* and *S* are enhanced simultaneously, whereas the annealed samples exhibit small decreases in *S* with relatively largely increased *σ*. For example, for the sample annealed at 473 K, *σ* increases by a factor of 1.7 and *S* only decreases by 10%. Therefore, the power factor increases by 35%, from 304 to 410 μV/K. The enhanced power factors are strikingly evident in Figure 5b.

**Figure 6:**
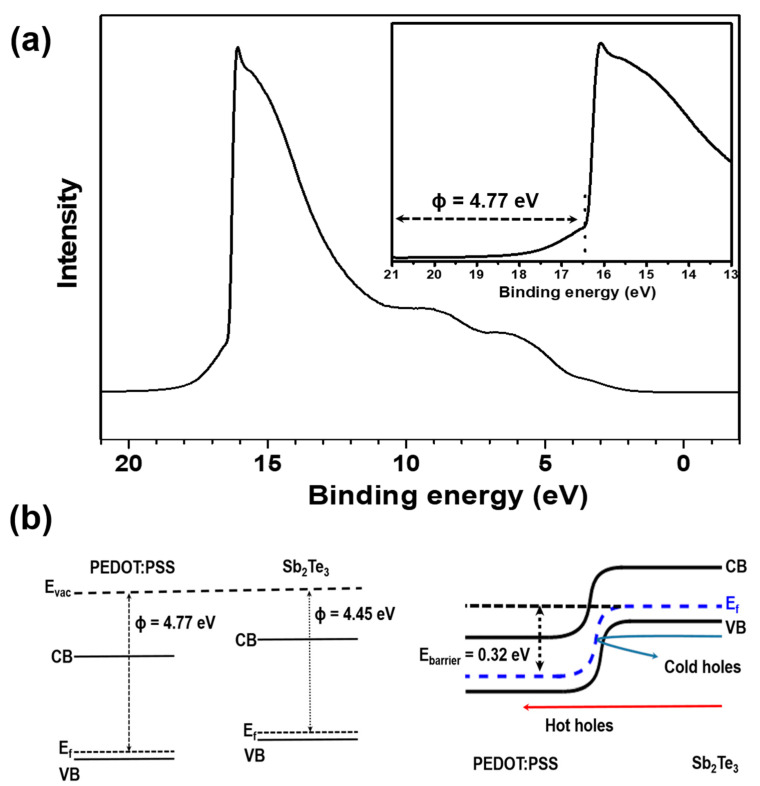
(**a**) UPS (ultraviolet photoelectron spectroscopy) spectrum of PEDOT:PSS-PVP nanofibers (the inset shows the calculated work function), and (**b**) band diagram at the interface between the Sb_2_Te_3_ and PEDOT:PSS-PVP.

Generally, a decrease in the *S* is accompanied by a *σ* increase due to increased carrier concentration. However, the *S* value can be enhanced even with *σ* increase either by the quantum confinement effect [37] or carrier energy filtering effect [38,39]. As the STPPST composite films exhibit higher thermoelectric power factors, it is likely that the embedded PEDOT:PSS-PVP nanofibers block low energy carriers by the potential barrier due to the percolation effect [38]. Thus, to examine the carrier energy filtering effect in the composite samples, a UPS spectrum was obtained (Figure 6a). The secondary cut-off of PEDOT:PSS was measured at 16.45 eV and the work function was calculated as 4.77 eV using Equation (1) [40]:

Φ = *hν* − *E*_cut-off_ (1)

where the *hν* is the energy of He(I) irradiation (21.22 eV) and *E*_cut-off_ is the secondary cut-off level. Thus, as depicted in Figure 6b, an energy barrier with a height of ~0.32 eV can be formed near the interface between PEDOT:PSS and Sb_2_Te_3_, while the work function of Sb_2_Te_3_ is 4.45 eV [41]. Most of the carriers can pass over the energy barrier, although low energy carriers are blocked. Therefore, the *S* can be increased because of the reduced coupling of electrons and holes in the valence band. The embedded PEDOT:PSS nanofibers formed in the Sb_2_Te_3_ matrix effectively transport charge carriers, and the potential energy barrier formed between the PEDOT:PSS and Sb_2_Te_3_ hybrid composite seems to block the low energy carriers around the Fermi energy level, according to the charge filtering effect.

## 4. Conclusions

The present work demonstrates the preparation of thermoelectric Sb_2_Te_3_/PEDOT:PSS-PVP/Sb_2_Te_3(ED)_ hybrid composite films via a stepwise, multi-coating method involving sputtering, electrospinning, and electrodeposition. The advantages of the STPPST hybrid composite films are seen in their stable preparation at room temperature and their immediate applicability for inorganic-organic composite-based thermoelectrics. The STPPST composite films will have applicability as electrode materials in thermoelectrics in the near future, and can be envisioned for use in heat-to-electrical-energy conversion systems. This work provides a smart approach to the design and modulation of the thermoelectric properties of conducting polymer/inorganic nanostructure composites.

## Figures and Tables

**Table 1:** *σ*, *S*, and power factor values of reference Sb_2_Te_3_/Sb_2_Te_3(ED)_ film and the Sb_2_Te_3_/PEDOT:PSS-PVP/Sb_2_Te_3(ED)_ films with different annealing conditions.

	Reference Sb_2_Te_3_/Sb_2_Te_3(ED)_			Sb_2_Te_3_/PEDOT:PSS-PVP/Sb_2_Te_3(ED)_		
	w/o Annealing	Annealed at 373 K	Annealed at 473 K	w/o Annealing	Annealed at 373 K	Annealed at 473 K
*σ* (S/cm)	18.7	82.7	186	21.3	135	318
*S* (μV/K)	160	134	128	164	121	114
Power factor (μW/mK^2^)	47.6	149	304	57.1	198	410
